# The Story of the Insane from Year to Year

**Published:** 1897-08-28

**Authors:** 


### THE STORY OF THE INSANE FROM YEAR TO YEAR.

Tenth Series.

### THE CITY OF LONDON ASYLUM AT STONE, DARTFORD.

There were 470 patients in residence on January 1st, 1896, and 485 on the last day of December. There were 106 first admissions, 7 readmissions, 46 recoveries, 23 discharged relieved, and 23 deaths. The recoveries stand at 46 per cent. of the admissions, and the deaths at 4·80 on the average number resident; the former is above the county asylum average and the latter much below it. Of the deaths 12 were caused by cerebral and spinal diseases and 1 by consumption. Turning to Table X. we find that 17 of the year's admissions owed their insanity to moral causes, such as domestic trouble, religion, and adverse circumstances, 18 to intemperance in drink, and 19 to hereditary influence. One special feature about this asylum is the number of private patients it receives. There were 70 in residence at the close of the year; 34 had been admitted, and 11 discharged recovered. Dr. White may well say that the success of this undertaking has exceeded his most sanguine expectations, and we may add that it points out the way to other counties, for there is no part of England where it would not be a source of profit to the asylum and a boon to the inhabitants. Dr. White concluded the ninth annual course of lectures to the attendants, and three of them obtained the certificate of the Medico-Psychological Association and also the silver badge presented by the asylum. New hospital wards and other additions are being put up, and these will make up for the defects in the old building. The farm shows a profit of £189 after deducting rent and taxes and the other usual charges. The visitors say that "the medical superintendent and the officers continue to discharge their duties with efficiency and zeal." The weekly cost of maintenance is 10s. 11½d. per head. Private patients pay £1 1s. a-week.

### WARNEFORD HOUSE, OXFORD.

There were 85 patients in residence on January 1st, 1896, and 92 on the last day of December. There were 18 admissions, 5 recoveries, and 3 deaths. When the numbers are so small, percentages are almost useless. For instance, the recoveries at the male side were only 11·1 per cent. of the admissions, whereas on the female side they reached 100 per cent. The deaths were 3·3 on the average number resident. Three of the admissions owed their insanity to intemperance in drink, and 10 to hereditary tendency, which means that 13 out of 18 admissions would never have existed at all provided due control had been exerted over appetites and sufficient care exercised in choosing wives and husbands. It is gratifying to learn that 71 of the 92 patients are received at rates lower than the actual cost, but it would be much more satisfactory if a table were given showing the various charges made for those patients who do not pay the full amount.

### CHARTRAM DOWN ASYLUM, KENT.

On December 31st this asylum contained 900 patients, having increased by 13 during the year. There were 155 first admissions, 12 readmissions, 55 recoveries, and 80 deaths. The recoveries stand at 33 per cent. on the admissions, and the deaths at 8·8 per cent on the average number resident. Of the deaths 34 were caused by cerebral diseases and 10 by consumption. Of the admissions 34 owed their insanity to various moral causes, while of the physical causes 40 are put down to hereditary influence and 32 to drink. The medical officers have continued to give lectures and instructions to the staff, and three of the attendants have obtained the nursing certificates, a number which Dr. Fitzgerald hopes to see increased. The weekly cost per head was 9s. 5⅝d.

### THE DONEGAL DISTRICT ASYLUM AT LETTERKENNY.

The limits of accommodation are given at 422 patients, but there were in residence on January 1st, 1896, 426 patients, and by the end of the year this number had risen to 461, which shows there was overcrowding to the extent of 39 patients. There were 95 first admissions, 36 readmissions, 33 recoveries, 17 discharged relieved, and 45 deaths. The recoveries were 25·2 per cent. on the admissions, and the deaths were 10·3 per cent. on the average number resident. Of the year's admissions 16 were due to moral causes, 6 to intemperance in drink, and 34 to hereditary influence. Of the deaths, 15 were caused by consumption, 2 to dysentery, and 1 to enteric fever. There were 10 cases of the latter disease. The water supply has for some time been deficient, but steps are now being taken to remedy this, perhaps the greatest, defect an asylum could have. The cost per head per annum is £21 19s. 6d.

### THE ROYAL ASYLUM, ABERDEEN.

The statistical tables show a small decrease in the numbers, there having been 741 patients in the asylum on January 1st, and 736 on the last day of the year. There were 152 first admissions, 55 readmissions, 95 discharged recovered, 33 discharged relieved, and 55 deaths. The recoveries reach the very creditable percentage of 45·9 on the admissions, and the deaths at 7·5 on the average number resident are lower than usual in public asylums. Of the deaths, 25 were caused by cerebral and spinal diseases, 9 by consumption, and 2 by enteritis. Domestic trouble, adverse circumstances, religion, &c., account for 23 of the admissions, intemperance in drink for 23, and hereditary influence for 91. Dr. Reid calls attention to two forms of insanity which are commoner out of asylums than in them. The first is known as moral insanity, because the moral faculties are affected, and the intellectual faculties but little, if at all, deranged; and the second is that in which the power of the will is deficient. "In health," says Dr. Reid, "every individual has a supply of self-controlling power which regulates his conduct. Some have more, some have less, of this power, and it is the duty of him who knows his share of it to be deficient to endeavour to preserve and reserve it, and to seek means to keep his impulses under control, so that he shall not further impair it." These words are applicable to a large class, and it would be well if they would sink deep into their minds, but, unfortunately, such people are generally little prone to act on good advice. The asylum contains nearly 250 private patients.

### THE BERKSHIRE ASYLUM, MOULSFORD.

There were 558 patients in residence on January 1st, 1896, and 568 on the last day of December. There were 82 first admissions, 16 readmissions, 32 recoveries, 7 discharged relieved, and 47 deaths. The recoveries were 30·8 per cent. of the admissions, and the deaths 8·4 per cent. of the average number resident. Fifteen of the deaths were caused by cerebral and spinal diseases, and 11 by consumption. Hereditary tendency accounts for 33 of the admissions, and drink for 8. Of late years nearly all our county asylums have been flooded with chronic cases sent in from the workhouses, and Dr. Murdoch truly says: "It is most discouraging to the medical and nursing staff to be confronted day by day with the admission of hopelessly incurable cases, consisting, for the most part, of the congenitally defective, epileptics, and aged," and he adds that "it is, perhaps, advisable to detain all the mentally-affected persons in one asylum; but this fact must be fully recognised by the public when the question of finding increased accommodation is forced upon those of their representatives who are responsible for the providing and maintaining of such public institutions." Dr. Murdoch arrives at the conclusion, comforting to the Berkshire residents, that there is no actual increase of insanity in the county, except what can be accounted for by increase in the population. The visitors express their "extreme satisfaction at the able manner the asylum has been managed by Dr. Murdoch." The average weekly cost per head was 7s. 5½d.

### THE NOTTINGHAM BOROUGH ASYLUM AT MAPPERLY HILL.

Here the numbers increased from 576 to 601. There were 146 first admissions, 22 readmissions, 65 recoveries, 6 discharged relieved, and 49 deaths. The recoveries stand at 39·9 per cent. of the admissions, and the deaths at 8·2 per cent. of the average number resident. Of the deaths 31 were caused by cerebral and spinal diseases, and one by consumption. Of the year's admissions 32 owed their insanity to various moral causes, the same number to intemperance in drink, and 12 to hereditary tendency, while in 28 no cause was ascertained. There is the same cry of deficiency of room at this asylum which we hear in nearly every county and borough in England, and with an overcrowding on the males' side to the extent of 20 patients and only four vacancies on the women's side of the house it is clear that something must be done. Mr. Powell has tried to relieve pressure by discharging chronic cases to the care of their relatives, but he finds that several of these have been returned to the asylum as being unmanageable at home, and this is a peculiarity by no means rarely met with; a patient who goes on quietly and harmlessly in an asylum becomes troublesome and dangerous when sent out. Indeed, the more one sees of chronic lunatics the less one trusts them. We are more than half inclined to agree with Mr. Powell when he says that the reasons usually given for the increase in the number of the insane in asylums are not wholly satisfactory to him, and he says he is "reluctantly obliged to come to the conclusion that in Nottingham there has undoubtedly been a substantial increase in the incidence of insanity in recent years." Mr. Powell continues his instructions to the staff and four attendants, and two nurses have obtained the Medico-Psychological Nursing certificate. The farm is credited with a balance of £294 in its favour, but rent of land, interest on capital, and patients' labour are not given, nor are the prices given at which farm produce is supplied to the asylum. The average weekly cost per head was 9s. 5d.

